# dfgcompare: a library to support process variant analysis through Markov models

**DOI:** 10.1186/s12911-021-01715-3

**Published:** 2021-12-20

**Authors:** Amin Jalali, Paul Johannesson, Erik Perjons, Ylva Askfors, Abdolazim Rezaei Kalladj, Tero Shemeikka, Anikó Vég

**Affiliations:** 1 Department of Computer and Systems Sciences (DSV), Stockholm University, 16407 Stockholm, Sweden; 2 Health and Medical Care Administration, Region Stockholm, 10431 Stockholm, Sweden

**Keywords:** Process variant analysis, Process mining, Markov chain

## Abstract

**Background:**

Data-driven process analysis is an important area that relies on software support. Process variant analysis is a sort of analysis technique in which analysts compare executed process variants, a.k.a. process cohorts. This comparison can help to identify insights for improving processes. There are a few software supports to enable process cohort comparison based on the frequencies of process activities and performance metrics. These metrics are effective in cohort analysis, but they cannot support cohort comparison based on the probability of transitions among states, which is an important enabler for cohort analysis in healthcare.

**Results:**

This paper defines an approach to compare process cohorts using Markov models. The approach is formalized, and it is implemented as an open-source python library, named dfgcompare. This library can be used by other researchers to compare process cohorts. The implementation is also used to compare caregivers’ behavior when prescribing drugs in the Stockholm Region. The result shows that the approach enables the comparison of process cohorts in practice.

**Conclusions:**

We conclude that dfgcompare supports identifying differences among process cohorts.

## Introduction

Process management is an important area that enables process improvement by saving cost, resources, and time [25]. The analysis of recorded data plays an important role in shifting process improvement from traditional approaches towards more data-driven approaches. Process Mining is a research area that enables process improvement by extracting information from logged data [47]. It enables discovering patterns and process models from the event log, checking the conformance of executed process instances with the regulations related to the organization at hand, and enhancing processes with the help of insights that can be identified from log files [47].

The enactment of healthcare processes results in large variations of process execution [25], i.e., process instances, which are recorded in event logs. Each of these variants is also known as a cohort [50]. It is usually not very helpful to discover a solo process model from such event logs since the discovered model includes scenarios for all cohorts. Thus, the model is complex and hard to understand, a.k.a., spaghetti model [47]. The reason is that the discovered model includes so many process variants, and the model will look like spaghetti, meaning that every activity will be connected to others. The solution is to slice and dice the event logs to discover different process models for each cohort.

The comparison of process models for different cohorts is not an easy task to be done manually. Instead, software support would be useful. However, there are few software that supports cohort comparison. Currently, support is provided based on the frequencies of process activities and performance metrics. These metrics are effective in cohort analysis, but they cannot support cohort state-transition models (a.k.a. Markov models) which are widely used in healthcare studies, e.g., [8, 39].

Therefore, this paper proposes a new approach and provides an open-source library of Python to enable this comparison, named dfgcompare. This library is used to compare two process variants in the drug prescription process, i.e., (i) when caregivers use the drug recommendation systems to investigate drug–drug interactions, and (ii) when caregivers did not perform such investigation. The result shows that our approach enables the comparison of different process cohorts. As the drug-prescription process is complex, and the data cannot be shared publicly, we defined a simple running example and generated artificial logs. Based on this, we demostrate and discuss our approach. This data is available for readers, and it enables them to test the library.

The remainder of this paper is structured as follows. “Background” section gives a background on cohort analysis in the healthcare domain. It also introduces control-flow based process discovery and cohort analysis in process mining. “Approach” section introduces the approach, and “Implementation” section elaborates on the implementation. “Result” section demonstrates how cohort analysis is supported and used in practice. “Conclusion” section concludes the paper.

## Background

This section gives a background on process mining by explaining the control-flow based process discovery using a running example. Then, it gives a brief background on the process variant analysis. Finally, it elaborates on the Markov chain model, and it summarizes related work on the use of the Markov chain model in process mining in the healthcare domain.

### Running example

Our running example is about a care process in a hospital where we treat both emergency and non-emergency cases. To keep the process simple, imagine that we have only five activities in this process, i.e., register patient (rp), read patient’s journal (rj), visit patient (vp), update the journal (uj), and operate patient (op). These activities can be executed in different orders for emergency and non-emergency cases which represent different cohorts. The execution result is recorded in the log file.

An example of sample rows that can be recorded in a log file is shown in Table 1. Each event log shall consist the case identifier, activity name, and the sequential order of their executions, which are presented as *Case ID*, *Activity*, and *Order* in our example. In addition, the event log can consist of more data. For example, we have an attribute which is called *Is Emergency* which defines if the case was an emergency case or not. The additional attributes can provide more insight into the executed process. For example, the *Is Emergency* indicates the process variation in our example, which can be used to split different process cohorts. An example of other additional attributes can be the caregiver who treated the patient, etc.

**Table 1:** An excerpt from a sample event log

Row number	Case ID	Activity	Order	Is emergency
1	1	Register patient (rp)	1	False
2	1	Read patient’s journal (rj)	2	False
3	1	Visit patient (vp)	3	False
4	1	Update the journal (uj)	4	False
5	1	Operate patient (op)	5	False
6	1	Update the journal (uj)	6	False
7	2	Register patient (rp)	1	True
8	2	Read patient’s journal (rj)	2	True
9	2	Operate patient (op)	3	True
...	...	...	...	...

### Process discovery

Process discovery is a process mining technique that enables extracting process models from event logs. This technique can be used to discover different process perspectives including control-flow. In this paper, we only focus on this perspective. The input for a discovery algorithm is an event log that captures events which have happened in the process, and the output is a process model.

**Fig. 1:**
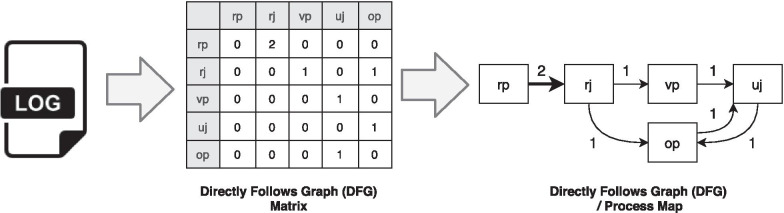
An overview of process discovery techniques

There are different ways to discover process models from event logs. One widely used technique is calculating the Directly-Follows Graphs (DFGs) matrix, which is considered as the de-facto standard for commercial process mining tools [48]. This approach is widely used since it is easy to use and implement. DFG based process discovery algorithms mostly has two steps, i.e., (i) calculating DFG matrix based on event logs, and (ii) visualizing DFG using the so-called process map. Figure 1 shows an overview of these steps.

The *first step* is calculating the DFG matrix by counting directly-follows relationships among events for different activity pairs in each case in the log files. In our figure, we only selected the first 9 rows which are shown in Table 1. The DFG matrix has a column and a row for each activity. The intersection cells of columns and rows store the number of directly-follows relationships for the corresponding activities, e.g., the intersection of the row *register patient (rp)* and column *read journal (rj)* shows the number of times that activity *rp* is followed directly by *rj* in the log file for different cases. As it can be seen in Table 1, *rp* is followed by *rj* twice, so the value for the intersection of *rp* row and *rj* column is set to 2 (see Fig. 1).

The *second step* is to visualize the process model. A simple representation of this matrix is a sort of process model called Directly-Follows Graphs (DFGs) or process map, which is represented on the right-side of Fig. 1. In this notation, nodes represent activities and flows represent their relations. It is common to set the thickness of each flow based on its frequency weight to visualize common paths.

### Process variant analysis

**Fig. 2:**
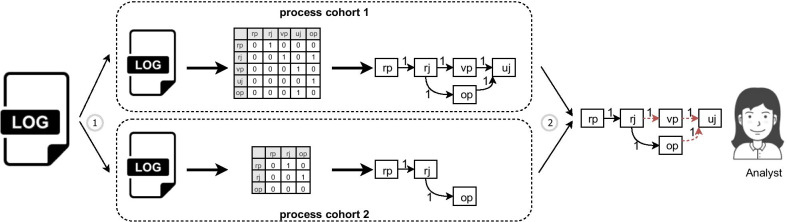
Process variant analysis

It is common to have different variations (cohorts) when running a process as different cases require different sorts of treatment, e.g., we have emergency and non-emergency cohorts in our running example. Process variant analysis is defined as a set of techniques that enable comparing different process variants based on information that is captured in the log file [46]. Figure 2 shows an overview of a process variant analysis technique using process maps.

A process variant analysis technique starts by splitting an event log into two sub-logs, each representing a cohort (marked by number 1 in Fig. 2). The split can be performed based on contextual data. Then, we can identify different process maps for each of the log files, as described before. Finally, we need to compare these two process maps using different metrics (like performance and frequency) to assist analysts to identify differences.

There are many studies that applied process variant analysis, which is reviewed recently by Taymouri et al. [46]. Among these studies, we only found three control-flow based process variant analysis software, i.e. Bolt et al. [11], Wynn et al. [50], Ballambettu et al. [7]. These are important contributions as they enable applying process variant analysis in practice. All these software support process variant analysis using frequency and performance metrics.

**Fig. 3:**
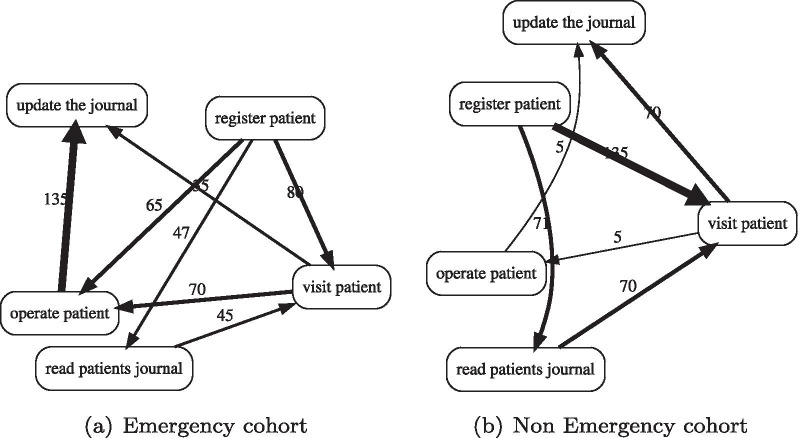
Example of process maps for different cohorts

The frequency and performance are not the only metrics based on which we can compare process cohorts. In healthcare domain, it is meaningful to compare cohorts based on the probability of state transition models, as reviewed in [44]. For example, we visualize two process maps for our emergency and non-emergency cohorts in Figure 3. We can compare these two cohorts using frequency or performance, but this cannot answer how much these two cohorts are different based on the probability that states can change in these models. The use of a Markov chain model can help us to solve this problem.

### Markov chain model

A Markov chain is a stochastic model that describes a process based on the probability of movement between different states, so nodes represent the status of a business process while flows represent the probability of movement from one state to another. Stochastic models have been used in the process mining area for different purposes, e.g., evaluating the quality of discovered process models [42] or conformance checking [31].

**Fig. 4:**
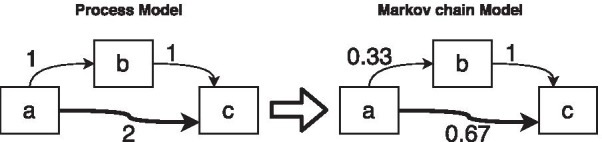
An overview of a Markov chain process

To explain Markov chains models, imagine that we discovered a DFG based on a log in which activities represent states of a business process. In this new setting, the discovered process model, as shown on the left-side of Fig. 4 shows the states of the process and the frequency of movement between states. The right side of Fig. 4 shows the same process model that is defined based on the Markov chain. The weight of each outgoing-flow for a state represents the probability of that flow given we are in the state of that activity in the process.

This is the basic idea behind the solution based on which we define our approach in the next section.

### Related work

This section presents how the Markov chain model is used in process mining in the healthcare domain. We searched for related work in two ways. First, we searched literature review papers in process mining in the healthcare domain, from which we selected research papers related to Markov models. Second, we searched research papers about Markov models in process mining in the healthcare domain. We used Scopus and Google Scholar as search databases in both searches. We limited the search using Title, Abstract, and Keywords. The search is done on 18th May 2021.

Through the first search, we used “Process mining” AND healthcare AND “literature review” as the search keyword. We excluded papers unrelated to the literature review of process mining in the healthcare domain from the result. Thus, we ended up with 7 papers, i.e., [21–23, 26, 30, 41, 49]. From these literature review papers, we identified 5 papers related to Markov models, i.e., [10, 13, 34, 38, 40]. Through the second search, we used “Process mining” AND healthcare AND markov as the search keyword based on which we found 4 papers among which 3 were peered reviewed, i.e., [3, 15, 51]. Here, we summarize these 8 papers that we found.

Cook and Wolf [13] are among the first authors who introduced process discovery. They introduced three methods to identify process models from event logs, i.e., (i) an algorithmic-based approach by adopting the Ktail method, (ii) a statistical-based approach by adopting the neural-network-based RNet method, and iii) a hybrid statistical and algorithmic approach by adopting the Markov method. They conclude that the Ktail and Markov methods are more promising.

Blum et al. discovered surgical workflows build using Hidden Markov Model and represented through a graphical user interface in [10]. Poelmans et al. [38] combined data and process discovery techniques to discover different process variants in the healthcare domain. They have used the combination of the Formal Concept Analysis (FCA) and Hidden Markov Models to identify outliers and discover process variants. These studies show the practical significance of the use of the Markov model in healthcare.

Rebuge and Ferreira [40] introduce a methodology to discover clusters of process variants from event logs and compare them through the Markov chain. This approach is useful when the process variants cannot be identified based on their data elements. They have evaluated their approach in healthcare through a case study for which they have implemented a tool. The tool is developed and customized for this specific case study. It is dependent on SQL Server 2008 Analysis Services. Although the tool is not available, this paper elaborates well on the methodology. It also demonstrates the importance and practical significance of comparing process variants through a healthcare case study.

Yang et al. [51] investigated how the Hidden Markov Model (HMM) can be used to discover process models. They also proposed a new approach, called Alignment-Guided State Splitting HMM interface algorithm (AGSS), to discover medical processes with better performance. Alharbi et al. [3] show how enriching event logs with Hidden Markov Models can facilitate process discovery by enabling analysts to deal with the complexity that exists in healthcare processes. Najjar et al. [34] also show how to analyze the complex and heterogeneous data in the healthcare domain with the help of the Markov models. Their approach enables the clustering of patient treatment pathways.

Oliveira [15] proposes a new methodology to deal with the complexity of medical event logs. This paper is appeared by searching “Process mining” AND healthcare AND markov in Abstracts in Google Scholar. The paper has no relation with Markov models, except it elaborates on related work. We realized that the paper is appeared in the search result due to having “Hidden Markov models” in IEEE Keywords.

To sum up, several case studies show the practical importance of using Markov models in the healthcare domain [3, 10, 15, 34, 40] among which [40] shows the importance of discovering process variants and the practical significance of comparing them. Currently, no tool is available to researchers to enable comparing process variants using Markov models.

## Approach

This section introduces the approach that we used to compare process variants. Our research is a Design Science research [29], as we aim to provide software that supports process variants comparisons using Markov models. This section provides formal definitions which are defined to support such comparison. First, it gives an overview of the approach as an informal introduction. Then, the formal definitions are given.

### Overview

To compare models for different variants, we can compare their DFGs. DFG is basically defined for events which are based on activities, but it can also be used to discover state-oriented processes if we map states as nodes and transitions as flows. Each event corresponds to one row in the event log, and it has information about the case identifier, state of the process and timestamp.

The frequency of flows in DFG might not be a good candidate for comparing two process variants. In our study, we realized that the probability of moving from one state to another is a better base for comparison, so we first convert the DFG to stochastic DFGs, based on which the Markov chain version of the process maps can be discovered. Moreover, the stochastical DFGs are a good base for comparison, because we can calculate the differences between two models based on different probability of moving from one state to similar states in the other model. To enable discovering significant differences, we also defined a cut off level, which ignores the difference if it is less than a threshold, which is the same as other process discovery algorithms like Fuzzy Miner [24].

### Formal definitions

Let’s start defining the formal definitions for Event Log and Directly Follows Graph (DFG) based on which we will define the rest of the algorithms.

#### **Definition 1**

(*Event Log*) An event log is a tuple $L=(E, S, C, \varepsilon )$, where:

- *E* is a set of Events,
- *S* is a set of states,
- *C* is a set of Cases,
- $\varepsilon :E\rightarrow (C, S, {\mathbb {N}})$ is a function that assigns a tuple of case, state and a natural number (as order) to an event, where:
  - $\#_c((c_1,s_1,n_1))=c_1$ denotes the case of the tuple $(c_1,s_1,n_1)\in (C, S, T)$,
  - $\#_s((c_1,s_1,n_1))=s_1$ denotes the state of the tuple $(c_1,s_1,n_1)\in (C, S, T)$,
  - $\#_n((c_1,s_1,n_1))=n_1$ denotes the order of the tuple $(c_1,s_1,n_1)\in (C, S, {\mathbb {N}})$,
- and $$E\cap S\cap C=\emptyset$$.

To explain these definitions, we can refer to the log file in Table 1. Imagine that the activity column in the log file represents the states, and each row in the log will represent an event. We refer to each event by $e_i$ where *i* represents the row number in the table.

- We can see nine events in this log file (each row represents one event), so $\{e_1,e_2,e_3,e_4,e_5,e_6,e_7,e_8,e_9\}\in E$.
- We can see two cases in Table 1, i.e., values 1 and 2 in Case ID column, so $\{1,2\}\in C$.
- We also can see six states in this log file (see Activity column), i.e., $\{rp,rj,vp,uj,op,uj\}\in S$.
- $\varepsilon (e_1)$ assigns (1, *rp*, 1) tuple to *e*1, meaning that this event is related to case number 1, register patient (rp) state, and with the order number 1.
- $\#_c(\varepsilon (e_1))$ represents the case element of $\varepsilon (e_1)$ tuple, which is the case of (1, *rp*, 1), which is 1—the first element of the tuple. In the same way, the state and order can also be retrieved.

#### **Definition 2**

(*DFG*) A DFG $\Psi \subseteq (S\times S\times {\mathbb {R}})$ is a set of state pairs and their frequency.

#### **Definition 3**

(*Stochastic DFG*) A stochastic DFG is a set of state pairs and the probability of transitions among each pair, i.e., $\Psi ^\prime \subseteq (S\times S\times \{x\in {\mathbb {R}}|0<x\le 1\})$.

To enable comparison of two variations of a business process, three steps need to be followed, i.e., (i) calculating DFGs for each variant, (ii) converting DFGs to stochastic DFGs, and (iii) comparing stochastic DFGs. The calculations for each of these steps are defined using the following algorithm.

*In the first step*, we calculate the DFGs of two variations in an event log. A DFG of an event log can be calculated by Algorithm 1, where it takes the event log and retrieves the DFG. This algorithm calculates the DFG by counting the number of occurrence for each two events of any state pairs that directly followed each other in the log for a case. 
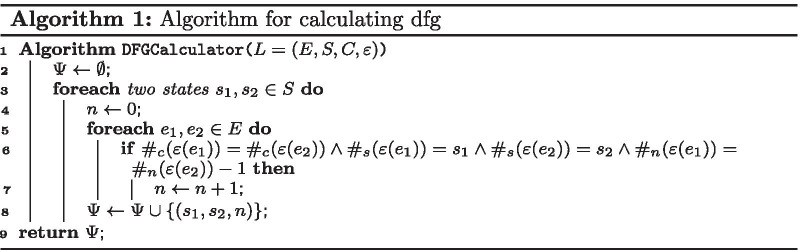


*In the second step*, we convert the DFGs to stochastic DFGs. The conversion of a DFG to a stochastic DFG is defined by Algorithm 2, where it takes the DFG and retrieves the stochastic DFG. This algorithm calculates the stochastic DFG by dividing the occurrences of each state pairs by the total occurrence of direct follows from the source state to all states. 
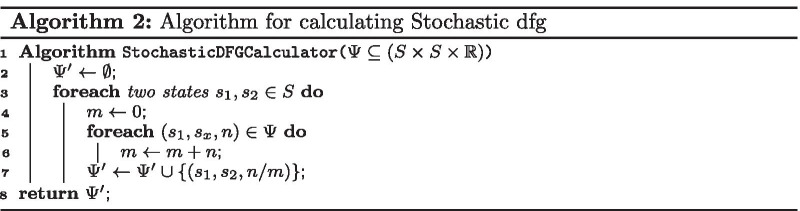


*In the third step*, we compare the difference between two stochastic DFGs. The comparison of two stochastic DFGs is defined by Algorithm 3, where it takes the stochastic DFGs in addition to a cut-off level and retrieves a stochastic DFG as a result. This algorithm calculates the difference for two stochastic DFGs by subtracting the values related to each corresponding state pair in the given stochastic DFGs. If the absolute value of the difference is greater than the cut-off level, it will include the difference in the result. 
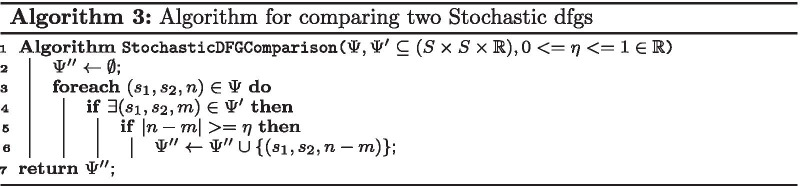


The final result is a DFG. To enable comparing positive and negative relations, we can visualzie flows with positive and negative weight using normal and dashed style respectively [45].

## Implementation

We have implemented our approach as a new library for Python, named dfgcompare. The library is implemented in Python because it is widely used by data scientists. There are a lot of libraries that also support data-driven analysis in Python. For example, Scikit-learn [36] supports different sorts of classification, regression, and clustering, or Tensorflow [1] supports analysis based on deep neural networks. There are also libraries to enable data-driven process analysis. For example, PM4Py [9] supports usual process mining algorithms or neo4pm that supports graph-based process mining [27].

We developed dfgcompare as an open-source project, which can be found on Github [16]. The library is also published in the Python Package Index (PyPI) [19], which is a public repository for python libraries. Thus, it can be used by all analysts who are using Python. We also defined a sample log based on our running example that can assist the analyst to explore the library. An example of such an analysis is also available on Github [17]. As a result, this library can easily be used by other researchers to compare process variants based on the Markov model.

## Result

This section demonstrates how the process variant analysis is supported by dfgcompare. First, the application of such analysis is demonstrated based on the running example. Then, we demonstarte how it is used in a healthcare project to recognize differences among two drug prescription cohorts.

### Demonstration on running example

**Fig. 5:**
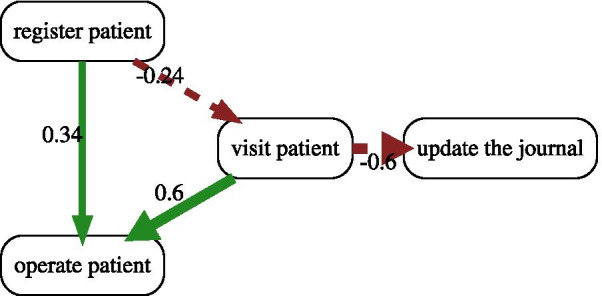
The comparision of process varients in the running example with cutoff 0.2

Figure 5 shows the comparison of the two process variants, i.e. emergency versus non-emergency cases, which are shown in Fig. 3. The cut-off is set to 20 percent meaning that the flows with more than 20% difference in probabilities are visualized. As can be seen, it is easy to compare the two variants now. In emergency cases, there is a 34 percent more chance to operate on patients directly after registration. Also, there is a 60% more chance to operate a patient after a visit. It is also more common to update the patient’s journal directly after visiting the patient in non-emergency cases.

### Case study

We evaluated our approach by comparing two varients in a drug-prescription process. To explain these variations, we need to elaborate on the interactions between drugs, called Drug–Drug Interactions (DDIs).

Drug–Drug Interactions (DDIs) can have serious side effects on patients [6], which not only can harm individuals but also result in a lot of cost on the healthcare system. Thus, there are several initiatives in defining systems that warn caregivers about these interactions when prescribing medicines, e.g., [6, 14, 28, 37, 43].

In the Stockholm region, a DDIs-aware system, called *Janusmed Interactions*, is integrated with the Electronic Health Record (EHR) systems that caregivers use to prescribe drugs [12]. It gives caregivers alert about possible interactions among drugs. The interactions are classified as A, B, C or D based on their clinical relevance [28], which are colored by green, white, yellow and red, respectively. If two drugs do not have any interactions, the system will not generate any alert. The system also provides detailed information about the reason for interactions, which can assist caregivers in investigating the interactions by clicking on the alert. The click will open the Janusmed website for the interaction.

**Fig. 6:**
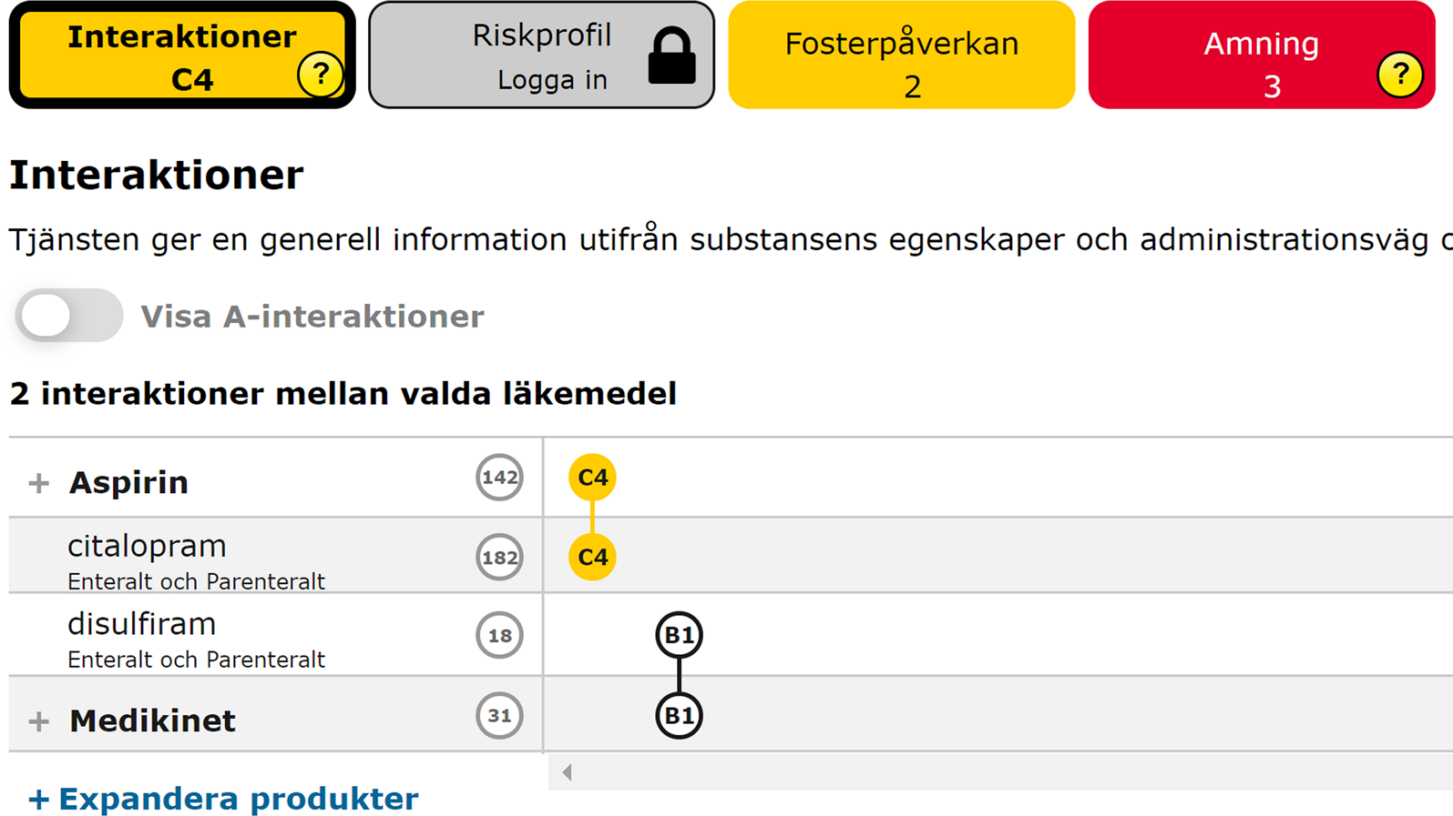
A screenshot of Janusmed system

Figure 6 shows a screenshot of the website through which caregivers can investigate the background of the alert. In this figure, two drugs have interaction C, while two others have interaction B.

The Janusmed system generates a huge amount of events per day. The logs include information about drug lists that were sent to Janusmed from the EHR system, which is needed for calculating the alert’s color. It records the state of the process, e.g., if the caregivers clicked on the alert, or the drug-list for which the alert is checked. Note that the process is sequential and there are no two activities that can be done in parallel in this process.

There are many studies on the use of DDIs support systems like Janusmed [5, 20], but there are very few data-based evaluations of such systems. One interesting aspect is to discover how caregivers reacted to different alerts and how the behavior of caregivers who investigated the background of alert is different from those who did not. Thus, we defined two variations, i.e. (i) care-givers clicked on the warning, and (ii) care-givers did not click on the warning.

### Data processing

**Fig. 7:**
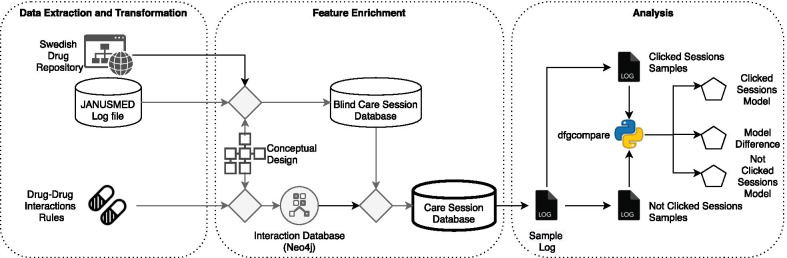
Data processing overview

The overall picture for Data Processing is shown in Fig. 7, which includes three sections, i.e., Data Extraction and Transformation, Feature Enrichment, and Analysis.

#### Data extraction and transformation

The main source of data in this project is *JANUSMED Log file*. It contains requests that have been received from different EHR systems by Janusmed for around 15 months. We name each request a *call*, and the log file contains around 155 million calls. Each call has information about the drug list, patients’ demography information, the EHR system, and much more information. We faced several challenges related to data.

As the first challenge, the data was incomplete, meaning that the log file did not capture the alerts given to caregivers. It also did not contain a case id for each session through which a patient was treated. Thus, we obtained information about *Drug–Drug Interactions Rules* as another source to reconstruct the alerts. Another challenge was that the drug code in the log was not compatible with the drug codes in the Drug–Drug Interactions Rules. Thus, we did web scraping to retrieve the drug codes from *Swedish Drug Repository*.

The incompatibility of the drug codes is rooted in different codings within the drug registration process. A drug shall follow the procedure that is defined by the Swedish eHealth Agency (eHälsomyndigheten) to be used in the Swedish market - like any other product [4]. In summary, different codes can be used for a drug depends on its status in the process. For example, NPL (Nationellt Produktregister för Läkemedel) is a national product registration code for drugs that are approved to be used in Sweden. This code does not give any information about the substances of the drugs. Our *Drug–Drug Interactions Rules* refers to drugs using their NPL code. It also contains the relation between drugs and their substances in addition to interactions that may exist among different substances.

The *JANUSMED Log file* that we have used in this study refers to drugs using svenskt godkännandenummer. We did not have the mapping of this code and NPL code, so we obtained the mapping of these codes by doing web scraping from *Swedish Drug Repository*. We could not simply do this for each drug in each call, as it was 155 million calls in our log file, each of which contains several drugs. Thus, we developed a caching layer to check if a mapping for a drug has been retrieved previously or not. This could reduce the number of requests that we sent to the *Swedish Drug Repository* significantly.

As a result, we ended up with three data sources for this project, i.e., JANUSMED Log file, Drug–Drug Interactions Rules, and Swedish Drug Repository, which made it possible to map the different code standards for drugs.

#### Feature enrichment

As we mentioned, our data sources lack the case identifier, and the drug–drug interaction alerts result. Thus, we enriched the features in our datasets through the second phase, called Feature Enrichment. To do so, we defined a conceptual model that specifies the relations between data elements in our business process.

As the second challenge, we defined the case identifier in our logs based on our conceptual design. Case identifiers are discovered by mapping features based on our conceptual design in the log files. As a limitation in this phase, we assumed that a case could not take over midnight for which we might have split some sessions into two sessions. The result is stored in a dataset, called *Blind Care Session Database*. In total, we ended up with around 24.5 million sessions. We verified the correctness of the case identification process using a random selection of cases.

As the third challenge, we realized that the logic behind interaction alerts are very complex. Thus, we created a graph database in Neo4j [35] based on our conceptual design and the Drug–Drug Interactions Rules. In this way, we can identify the alerts result that was generated for each call. Thus, we enriched the data in the Blind Care Session Database and generated another data set, called *Care Session Database*.

The feature enrichment was a very time-consuming process, in which we had to discover the interactions between drugs in 155 million calls. Therefore, we developed our data processing toolkit, which is a sort of light workflow engine. This toolkit enables us to execute different steps. It also supports resuming the process if a step fails, which enabled us to develop this process step by step. The use of a workflow engine was essential in feature enrichment; otherwise, it would be challenging to handle such a long-running cleaning process at once. In the end, our toolkit saved us a lot of time and enabled us to deal with the complexity of data processing. However, we realized that the data cleaning process would have been more efficient if we have used some currently available libraries, e.g., Luigi [32] or Apache Airflow [2]. They could reduce our development and maintenance time further, which was an important lesson that we learned.

#### Analysis

The aim of this phase is to analyze the difference between the behavior of caregivers who investigated interactions, by clicking on the Janusmed Interaction system and read more about the interaction, and those who did not.

As the fourth challenge, we realized that the definition of states could be defined based on different features, which can enable us to define different sorts of business process models from different perspectives. For example, it can be considered to define the state as if caregivers received different sorts of alerts or checked the website for the received alerts. The other possibility is to define states at a more granular level, which includes drugs as well. The process will be completely different in this case and will be more complex. This is common in applying process mining in practice as there is different information about a business process based on which different process maps can be identified. For the aim of this paper, we defined the states based on DDIs results and whether the care-givers clicked on the warning to check the website.

As the fifth challenge, we faced the problem to analyze the whole data due to its huge amount. Note that each call resulted in many rows since they have many drugs, and each drug can have many substances. Thus, we write programs that helped us to get an idea of overall trends in our dataset. We realized that the number of sessions for working days are very different in comparison with non-working days. Thus, we considered this finding in our sampling.

We performed stratified sampling by considering the weight of working and non-working days and collected traces randomly to analyze our data. We defined a program that performed the random stratified sampling, and the result is stored in *Sample Log*. The sample log is split into two categories, i.e., Clicked Sessions Samples and Not Clicked Sessions Samples. This technique is called the drilling down use a case in the process mining healthcare reference model [33]. The *Clicked Sessions Samples* contains traces when the caregiver clicked on drug–drug interactions alerts at least once, while the *Not Clicked Sessions Samples* contains all other traces.

**Fig. 8:**
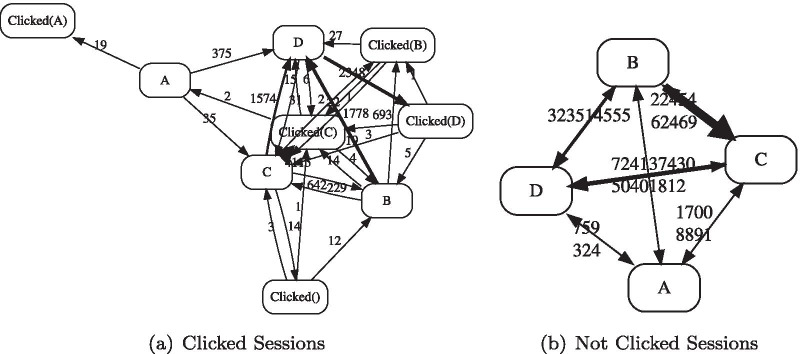
Process maps for the two process variations

**Fig. 9:**
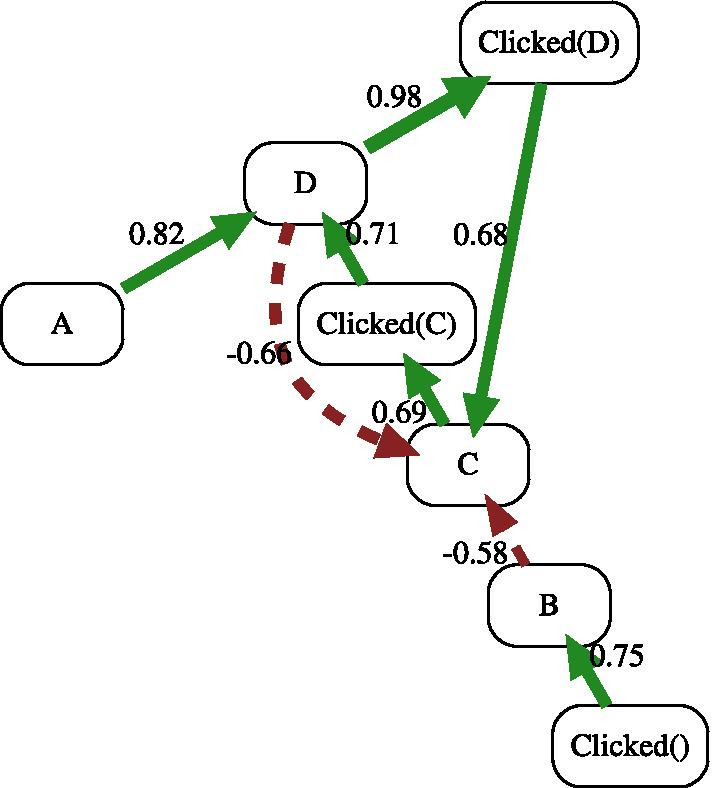
Comparisons of models with cut-off 0.5

We used dfgcompare through which we discovered two stochastic DFGs, shown in Fig. 8. The left- and right-sides of Fig. 8 show the DFG (process map) for Clicked Sessions sample and Not Clicked Sessions sample, respectively. The comparison of these two processes is a very cumbersome task if it shall be done manually. We used the dfgcompare to compare these two Stochastic DFGs. Figure 9 shows the comparison result with cut-off level of 50%. This process map compares the clicked session variants versus not clicked sessions ones. We can identify some interesting patterns from this comparison:

- $A \overset{0.82}{\longrightarrow } D \overset{0.98}{\longrightarrow } Clicked(D)$: It is more common that care-givers check the drug interactions warning when they face the most severe warning,i.e., D level. As it can be seen, it is 82 percent more probable that care-givers receive warning D after warning A in the clicked variant versus. non-clicked one. The reason can be that care-givers check the warning in case of facing a D-level warning, which can also be observed in this model as it is 98 percent probable that they do so. This means that there are few transitions from A to D in the non-clicked process variants.
- $C \overset{0.69}{\longrightarrow } Clicked(C) \overset{0.71}{\longrightarrow } D$: It is 69% more chance that care-givers check the C-level warning in clicked sessions variants than other path from this state. It is interesting the next state is D-level warning, meaning that they increased the warning level by adding drugs. This is common as they may try different drugs to check the one that has the least level of interactions.
- $B \overset{-0.58}{\dashrightarrow } C$: This is an observation that not sounds very good in running the healthcare process. It is 58% more chance that caregivers who received B warning add another drug and increase the level of drug–drug interactions but do not click to investigate the warning. Note that it is still possible that they have deleted the drug eventually as they might know the reason behind the interaction and did not need to investigate it.
- $D \overset{0.98}{\longrightarrow } Clicked(D) \overset{0.68}{\longrightarrow } C$ versus $D \overset{-0.66}{\dashrightarrow } C$: This is an interesting observation that shows care-givers are very careful about D-level interactions in both variants. As can be seen, in these two variants, caregivers reduced the interaction level to C by removing the interactive drug from the drug list.

The relevance of the result is validated by domain experts who are last four authors in this paper and who are responsible for operationalizing the drug–drug interaction system in Stockholm.

### Threats to validity

There is one threat to validity in our case study, which is adding case identifiers based on other attributes. We found the risk to have the wrong case identifier very minimum as the chosen attributes were very specific, e.g. considering the date, healthcare unit, IP address, age, sex, and other patients attributes to identify each case. To minimize the risk, we did manual random verification where we could not find any problem in case identifiers. In case of such noise, the impact will be very minimal as we consider the probabilities in a very large sample data.

## Conclusion

This paper proposes a new approach for process variant analysis. The approach is defined by converting Directly-Follows Graphs (DFGs) for each variant into stochastic DFGs based on Markov models. The process comparison is then defined based on stochastic DFGs, which enables cohort comparison based on the probability of transitions among states rather than the frequencies of process activities and performance metrics. Therefore, it can support comparing process cohorts based on probability differences when moving between different process states. The approach is formalized and implemented as an open-source library in Python. The approach is demonstrated through a fictitious running example. It is also evaluated through a healthcare case study.

In the case study, we investigated how the drug prescription process is different based on two variations. The first variation is related to the process when caregivers use the drug recommendation systems to investigate drug–drug interactions, while the second variation is about the process that caregivers did not perform such investigation. The result shows that our approach enables comparing these two variants in the drug prescription process.

We provided the software support for process variants comparison using Markov models based on requirements that we found in literature and the study context. Theoretically, it is possible to extend this support by considering other theoretical aspects like the notation of distance between Markov models.

This study shows the practical significance of comparing process variants through Markov models. The practical significance of the comparison was that it supported business analysts in identifying differences between the process variants, thereby allowing them to identify differences in the behavior of different groups of process participants. In our case study, one group consisted of caregivers who carefully checked the details of interactions on the Janusmed website, and another group consisted of caregivers that did not do so. The difference between these two groups’ behavior enabled us to compare how different process participants actually used the system. This comparison can raise potential points for improving the system in the future.

## Availability and requirements

The source code for the dfgcompare library and running example data are available. Here are details to access the files:

- Project name: Process Mining Janusmed
- Project home page: https://processminingjanusmed.blogs.dsv.su.se/
- Programming language: Python
- dfgcompare Source Code on GitHub [16]
- dfgcompare running example on GitHub [17]
- dfgcompare on the Python Package Index [19] (“pip install dfgcompare”).
- Operating systems: Platform independent core (tested under Linux, Windows 10, Windows server 2019).
- Other requirements: graphviz, pandas. This package has dependency to graphviz, so the installation guideline shall be followed as described in [18]
- License: MIT License.
- Any restrictions to use by non-academics: None

## Data Availability

The test datasets which is used to demonstrate the tool is available through the dfgcompare package [19]. The sample code to retrieve the data is available at [17].

## Abbreviations

DFG: Directly-follows graph
DDI: Drug–Drug interaction
EHR: Electronic health record
